# Dissemination Bias in Systematic Reviews of Animal Research: A Systematic Review

**DOI:** 10.1371/journal.pone.0116016

**Published:** 2014-12-26

**Authors:** Katharina F. Mueller, Matthias Briel, Daniel Strech, Joerg J. Meerpohl, Britta Lang, Edith Motschall, Viktoria Gloy, Francois Lamontagne, Dirk Bassler

**Affiliations:** 1 Center for Pediatric Clinical Studies, University Children's Hospital Tuebingen, Tuebingen, Germany; 2 Basel Institute for Clinical Epidemiology and Biostatistics, University Hospital Basel, Basel, Switzerland; 3 Department of Clinical Epidemiology and Biostatistics, McMaster University, Hamilton, Canada; 4 CELLS – Center for Ethics and Law in Life Sciences, University of Hannover, Hannover, Germany; 5 German Cochrane Centre, Medical Center - University of Freiburg, Freiburg, Germany; 6 Center for Medical Biometry and Medical Informatics, Medical Center – University of Freiburg, Freiburg, Germany; 7 Clinical Research Centre Clinique du Centre Hospitalier Universitaire de Sherbrooke, Université de Sherbrooke, Sherbrooke, Canada; 8 Department of Neonatology, University Hospital Zuerich, Zuerich, Switzerland; University of Chieti, Italy

## Abstract

**Background:**

Systematic reviews of preclinical studies, *in vivo* animal experiments in particular, can influence clinical research and thus even clinical care. Dissemination bias, selective dissemination of positive or significant results, is one of the major threats to validity in systematic reviews also in the realm of animal studies. We conducted a systematic review to determine the number of published systematic reviews of animal studies until present, to investigate their methodological features especially with respect to assessment of dissemination bias, and to investigate the citation of preclinical systematic reviews on clinical research.

**Methods:**

Eligible studies for this systematic review constitute systematic reviews that summarize *in vivo* animal experiments whose results could be interpreted as applicable to clinical care. We systematically searched Ovid Medline, Embase, ToxNet, and ScienceDirect from 1^st^ January 2009 to 9^th^ January 2013 for eligible systematic reviews without language restrictions. Furthermore we included articles from two previous systematic reviews by Peters et al. and Korevaar et al.

**Results:**

The literature search and screening process resulted in 512 included full text articles. We found an increasing number of published preclinical systematic reviews over time. The methodological quality of preclinical systematic reviews was low. The majority of preclinical systematic reviews did not assess methodological quality of the included studies (71%), nor did they assess heterogeneity (81%) or dissemination bias (87%). Statistics quantifying the importance of clinical research citing systematic reviews of animal studies showed that clinical studies referred to the preclinical research mainly to justify their study or a future study (76%).

**Discussion:**

Preclinical systematic reviews may have an influence on clinical research but their methodological quality frequently remains low. Therefore, systematic reviews of animal research should be critically appraised before translating them to a clinical context.

## Introduction

Preclinical research has its main purpose in enhancing our understanding of physiologic and pathologic processes. However, preclinical studies, *in vivo* animal experiments in particular, also influence clinical research and might thus even influence clinical care by i) informing the design of clinical studies, ii) informing clinical guidelines that consider preclinical evidence when clinical evidence is lacking, or iii) directly guiding clinical practice. But the benefit of animal research on humans has been questioned [1], [2].

Systematic reviews offer a systematic and transparent way to comprehensively identify, evaluate, and critically appraise available evidence on a specific topic. Meta-analyses increase precision and generalizability of effect estimates by quantitatively summarizing the results of individual studies included in a systematic review in order to provide a single best estimate with maximal statistical power [3]. Systematic reviews and meta-analyses of preclinical studies are still relatively rare in the medical literature: Mignini et al. identified 30 systematic reviews of laboratory animal experiments in 2006 and Peters et al. found 86 using a more sensitive search strategy and a broader definition of laboratory animal experiments [4], [5]. But preclinical systematic reviews are getting more prevelant over the last years, as shown by Korevaar et al. in 2011 [6].

Methodological quality of primary animal studies is often not satisfying [7]. The Animal Research: Reporting of In Vivo Experiments (ARRIVE) guidelines for reporting animal research have been compiled to help improve the reporting of in vivo animal experiments [8], [9]. Apart from the poor methodological quality of primary studies, also the often low methodological quality of systematic reviews and meta-analyses of preclinical research can be problematic. While principles of critically appraising in systematic reviews of clinical research are well established [10], their application to systematic reviews of preclinical studies appears variable. Since 2004 the Collaborative Approach to Meta-Analysis and Review of Animal Data in Experimental Studies (CAMARADES) provides support for groups conducting systematic reviews and meta-analyses of data from experimental animal studies [11]. Some of their focuses include identifying potential sources of bias in animal work, developing recommendations for improvements in the design and reporting of animal studies, and developing better methodologies for meta-analysis of animal studies.

One of the major threats to systematic reviews is dissemination bias. Dissemination bias, often also referred to as publication bias, describes the selective publication and dissemination of results [12], [13]. In this situation, published studies are no longer a random sample of all studies that have been conducted, but constitute a biased sample leading to spurious conclusions. A recently published survey conducted in animal laboratories in the Netherlands reported that researchers (n = 454) thought that just about 50% of animal experiments are published and employees (n = 21) of for-profit organizations estimated that only 10% are published [14]. Lack of statistical significance was discussed as one of several important reasons for non-publication. Since the number of systematic reviews of preclinical research is growing, also the problem of dissemination bias in systematic reviews of preclinical research is getting more important [15]–[17]. But still dissemination bias is rarely considered in preclinical reviews. Peters et al. showed that only 37% (17/46) of meta-analyses considered dissemination bias [5], likewise, Mignini et al. reported that it has been considered only in 16% (5/30) [4]. Korevaar et al. reported that between 2005 and 2010 the proportion of meta-analyses of *in vivo* animal studies that assessed dissemination bias increased to 60% (21/35) [6]. Korevaar et al. completed their search for systematic reviews of animal experiments in 2009/10 [6].

Since systematic reviews of preclinical research are only now becoming more prevalent and new guidelines and support, such as CAMARADES are only recently becoming available, an update of the previous research to assess the development of systematic reviews of preclinical studies of the last years is crucial. Especially if one considers that preclinical systematic reviews may also influence clinical care it is indispensable to assess their methodological rigor not only to prevent unnecessary studies on animals but also on humans and eventually even unnecessary or in the worst case dangerous treatment of patients. Until today, the influence of preclinical systematic reviews on studies with human participants has not been evaluated. In this systematic review we will do a first step by analyzing the citation profiles of preclinical systematic reviews as a measure of the influence on clinical research.

This systematic review is part of the OPEN Project (*To* ***O*** *vercome failure to* ***P*** *ublish n* ***E*** *gative fi* ***N*** *dings*), which was designed with the goal of elucidating the scope of dissemination bias and non-publication of studies through a series of systematic reviews and policy evaluations (www.open-project.eu).

### Objectives

The specific goals of the present systematic review of animal studies are:

- To determine the number of published systematic reviews of animal studies until present.
- To investigate methodological features of systematic reviews of animal studies especially with respect to assessment of dissemination bias.
- To investigate the influence of systematic reviews of animal studies on clinical research by examining citations of systematic reviews by clinical studies.

## Methods

A detailed protocol of our methods has been published [18]. In brief, the following methods were used for the systematic literature search.

### Eligibility criteria

We used the same criteria as Peters et al. and Korevaar et al. [5], [6] and combined the results of our literature search (2009–2013) to the list of systematic reviews included in these previous works.

#### Inclusion criteria

We included systematic reviews and meta-analyses with a potential for being interpreted as applicable to humans.

The potential for being interpreted as applicable to humans was defined by the use of *in vivo* models and a focus on one of the following: i) the efficacy of a medical or surgical intervention, ii) the side-effects or toxicity of a medical intervention, iii) the mechanisms of action of a medical intervention, iv) risk factors for a human illness, v) the effects of an exposure to a chemical substance, vi) overview of animal models for disease, vii) the accuracy of diagnostic tests [6].

We defined systematic reviews as publications that described the source(s) searched for evidence as well as one of the following: i) the search terms used, ii) any limitation placed on the search, iii) explicit inclusion and exclusion criteria [5].

An article was included if it fulfilled one of these criteria of a broad definition of systematic review. Additional to this definition, as it has been used by Peters et al. [5] and Korevaar et al. [6], we used a second more stringent definition of systematic reviews. For the more stringent definition systematic reviews had to incorporate:

- a systematic search (statement on the search strategy, including more than one database, and “search terms” mentioned),
- explicit inclusion and exclusion criteria (statement of inclusion and exclusion criteria in the methods section),
- a focused research question (according to PICO (a technique used in evidence based practice to frame and answer a clinical question, or to develop literature search strategies, the acronym stands for Patient/Population Intervention Comparison/Control Outcome)) [19],
- a systematic evaluation of the risk of bias in included studies [5], [6], [10].

We define meta-analyses as publications incorporating a quantitative synthesis of results from animal experiments.

#### Exclusion criteria

We excluded genome-wide association studies and animal experiments with the main purpose to learn more about fundamental biology, physical functioning or behavior and not to inform human health-care. We did not exclude publications that incorporated results of clinical studies [6].

### Search strategy

We updated the search of Peters et al. and Korevaar et al. and therefore systematically searched electronic databases, Ovid Medline, Embase, Toxnet (http://toxnet.nlm.nih.gov/; including Toxline, DART, and HSDB) and ScienceDirect, all from 1^st^ January 2009 to 9^th^ January 2013 (the full search strategy is displayed in the study protocol [18]) [5], [6]. In addition, the bibliographies of any eligible articles identified were checked for additional references. No language restrictions were applied. We did not search any grey literature (eg literature that has not been formally published as journal articles).

### Study selection

Two reviewers, independently and in duplicate, screened titles and abstracts of search results. If a title and abstract could not be rejected with certainty by both reviewers, the full text of the paper was retrieved and assessed for eligibility. Any disagreement among reviewers was resolved by discussion and consensus or, if needed, third party arbitration.

### Data extraction

Working in teams of two, we independently extracted the following information from each eligible article (from this literature search and from the included articles by Peters et al. and Korevaar et al.): search strategy (database, language restriction, search of grey literature), clearly defined inclusion and exclusion criteria, list of included an excluded articles, formal assessment of methodological quality of included studies (by score (eg Jadad), by dimension (eg allocation concealment, blinding etc.), funding sources from included studies, report of a meta-analysis (report of effect estimates of individual studies, method for data synthesis), assessment of heterogeneity (Cochrane Q, I^2^, Tau^2^, other), assessment of dissemination bias (Funnel plot, Begg's or Egger's test, Fail-Safe Number, Trim and fill method, other).

Any disagreement was resolved by discussion and consensus or, if needed, third party arbitration.

### Appraisal of methodological quality of included reviews

We assessed the methodological quality of the included systematic reviews by focusing on various methodological features, such as clearly defined inclusion criteria, assessment of heterogeneity, assessment of dissemination bias, report according to guidelines. We did not use a scoring approach to assess the methodological quality.

### Data analysis and reporting

Data synthesis involved a descriptive summary of included studies.

### Investigation of the citation of systematic reviews of animal studies on clinical research

We used the Web of Science Internet-based citation database to identify clinical publications citing included systematic reviews and meta-analyses. We conducted this analysis in two randomly selected samples of included studies published between 2005 and 2009 to allow a minimum of 4 years to elapse between publication of the review and our analysis: we included 25 systematic reviews with a meta-analysis out of 29 and a random sample of 25 systematic reviews without a meta-analysis out of 57. We searched Web of Science on 11 August 2013 for clinical human studies or guidelines citing the selected animal reviews. All included studies were reviewed independently and in duplicate. The reviewers determined how the review of preclinical studies has been cited by the clinical study by allocating each citation to one of the following categories: i) used citation unrelated to animal studies in review, ii) used citation to provide at least partial justification for the study or a future study, iii) used citation to support or explain their findings, iv) used citation to discuss physiological pathways, and v) used citation to justify the measurement etc.

## Results

### Study selection and characteristics

**Figure 1:**
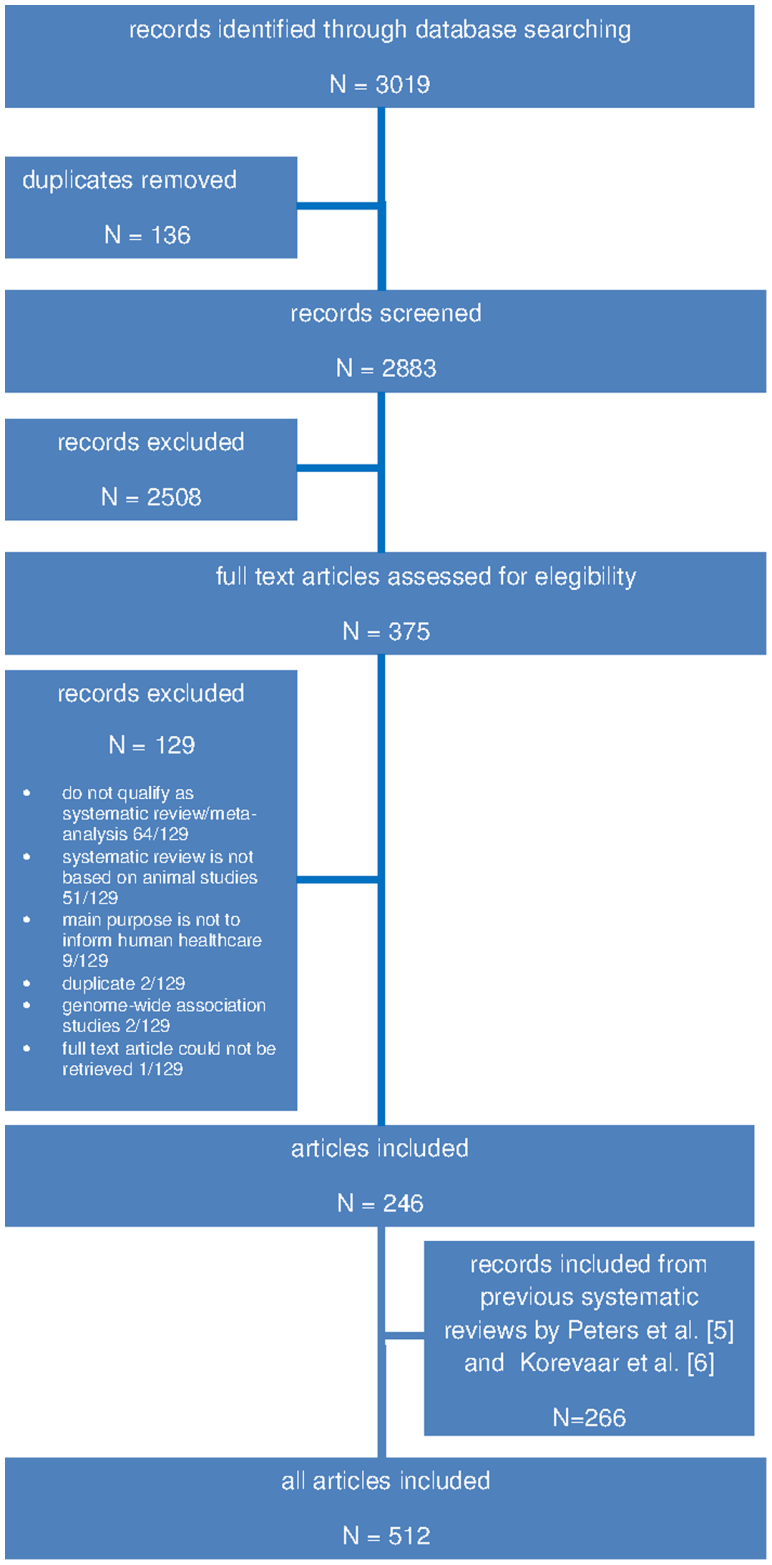
Flow chart for selection of systematic reviews included in the systematic review.

The literature search identified 3019 records. After screening titles and abstracts, we retrieved 375 full text articles and ultimately included 246 publications. Furthermore we augmented the list of included publications with the previous work by Peters et al. [5] (103 studies) and Korevaar et al. [6] (163 studies) (see Fig. 1). This shows an increasing number of published systematic reviews and meta-analyses on animal studies, a trend that had already been found in the two previous systematic reviews by Peters et al. [5] and Korevaar et al. [6].

512 of the included articles fulfilled the broad definition of a systematic review, but only 126 matched the more stringent definition of a systematic review. Most articles were excluded, because they did not qualify as systematic review or meta-analysis (50%). The objectives of the included systematic reviews and meta-analyses were mainly to investigate the efficacy of a medical or surgical intervention (41%). Additional characteristics of the included 512 systematic reviews (combined results from our literature search and the results of Peters et al. and Korevaar et al.) are summarized in Table 1.

**Table 1:** Characteristics of included systematic reviews.

	Results from Peters et al. and Korevaar et al.		Results from this literature search		All results	
	Number absolute	Number in percentage*	Number absolute	Number in percentage	Number absolute	Number in percentage
**Inclusion criteria**						
Broad definition of systematic review applied	266/266	100%	246/246	100%	512/512	100%
Narrow definition of systematic review applied	59/266	22%	67/246	27%	126/512	25%
**Objectives**						
i) investigation of the efficacy of a medical or surgical intervention	103/266	39%	108/246	44%	211/512	41%
ii) investigation of the side-effects or toxicity of a medical intervention	22/266	8%	20/246	8%	42/512	8%
iii) investigation of the mechanisms of action of a medical intervention	56/266	21%	46/246	19%	102/512	20%
iv) investigation of risk factors (epidemiological associations or mechanisms of action of disease)	8/266	3%	8/246	3%	16/512	3%
v) investigation of effects of an exposure to a chemical substance	20/266	8%	8/246	3%	28/512	6%
vi) overview of animal models for disease	50/266	19%	51/246	21%	101/512	20%
vii) investigation of diagnostic test accuracy	7/266	3%	5/246	2%	12/512	2%
**Type of Article (as presented in title or abstract)**						
Article presents itself as systematic review	194/266	73%	179/246	73%	373/512	73%
Article presents itself as meta-analysis	44/266	17%	20/246	8%	64/512	13%
Article presents itself as both	26/266	10%	31/246	13%	57/512	11%
Article presents itself neither as systematic review nor as meta-analysis	2/266	1%	16/246	7%	18/512	4%
**Literature search within review**						
Databases searched**						
Embase	76/266	29%	96/246	39%	172/512	34%
Toxnet	9/266	3%	6/246	2%	15/512	3%
Web of Science	8/266	3%	44/246	18%	52/512	10%
Medline	237/266	89%	239/246	97%	476/512	93%
Other	117/266	44%	137/246	56%	254/512	50%
Language restrictions applied						
Yes	92/266	35%	110/246	45%	202/512	39%
No	35/266	13%	43/246	18%	78/512	15%
Not reported	139/266	52%	93/246	38%	232/512	45%
Any attempt to search grey literature						
Yes	76/266	29%	63/246	26%	139/512	27%
No	24/266	9%	60/246	24%	84/512	16%
Not reported	166/266	62%	123/246	50%	289/512	56%
**Funding**						
Funding source extracted from included studies and reported	8/266	3%	6/246	2%	14/512	3%
Funding of the systematic review						
Not reported	168/266	63%	147/246	60%	315/512	62%
Governmental/public	56/266	21%	42/246	17%	98/512	19%
Industry/private for profit	11/266	4%	15/246	6%	26/512	5%
Charity/private not for profit	5/266	2%	13/246	5%	18/512	4%
Not funded/only in house source	26/266	10%	29/246	12%	55/512	11%

*all percentages rounded to integral numbers.

**multiple selection possible.

### Methodological features of included systematic reviews and assessment of dissemination bias

Only 59% of all the included systematic reviews clearly defined inclusion and exclusion criteria, and only just over half (51%) of the included studies displayed a list or flow diagram of the included studies, as suggested by the Preferred Reporting Items for Systematic reviews and Meta-Analyses (PRISMA) reporting guideline [20]. 24% of all the included studies did not report how many studies they included in their systematic review or meta-analysis. The majority of the included systematic reviews and meta-analyses of animal studies did not assess methodological quality of included studies (71%), nor did they asses heterogeneity (81%), or dissemination bias (87%). For more details, see Table 2.

**Table 2:** Methodological features of included systematic reviews.

	Results from Peters et al. and Korevaar et al.		Results from this literature search		All results	
	Number absolute	Number in percentage*	Number absolute	Number in percentage	Number absolute	Number in percentage
**Inclusion**						
Clearly defined eligibility criteria	145/266	56%	159/246	65%	304/512	59%
Number of included studies						
Not reported	79/266	30%	45/246	18%	124/512	24%
<10	23/266	9%	28/246	11%	51/512	10%
10–50	97/266	37%	103/246	42%	200/512	39%
51–100	36/266	14%	40/246	16%	76/512	15%
>100	31/266	12%	30/246	12%	61/512	12%
List/flow diagram of screened and included studies	111/266	42%	152/246	62%	263/512	51%
**Assessment of methodological quality**						
Not assessed	192/266	72%	171/246	70%	363/512	71%
Assessed by dimension	14/74	19%	22/75	29%	36/149	24%
Assessed by score	18/74	24%	20/75	27%	38/149	25%
Assessed differently	42/74	57%	33/75	44%	75/149	50%
**Assessment of heterogeneity**						
Not assessed	219/266	82%	197/246	80%	416/512	81%
Assessed by **						
Cochrane Q	4/47	9%	19/49	39%	24/96	25%
I^2^	8/47	17%	30/49	8%	38/96	40%
Tau^2^	2/47	4%	4/49	8%	6/96	6%
Other	29/47	62%	19/49	39%	48/96	50%
Assessed, but not described how	8/47	17%	3/49	6%	11/96	11%
**Dissemination Bias**						
Dissemination Bias mentioned	53/266	20%	60/246	24%	113/512	22%
Dissemination Bias assessed with **	29/266	11%	35/246	14%	64/512	13%
Funnel plot only	5/29	17%	12/35	34%	17/64	27%
Funnel plot and statistical test	4/29	14%	9/35	26%	13/64	20%
Begg's or Egger's test	13/29	45%	12/35	34%	25/64	39%
Trim and fill	2/29	7%	6/35	17%	8/64	13%
Fail Safe Number	3/29	10%	2/35	6%	5/64	8%
Other	2/29	7%	1/35	3%	3/64	5%
Evidence for dissemination bias	18/29	62%	14/35	40%	32/64	50%
**Reporting Guidelines (PRISMA, QUOROM) mentioned**	7/266	3%	34/246	14%	41/512	8%

*all percentages rounded to integral numbers.

**multiple selection possible.

### Results of included Meta-Analyses

In 31% of all the included studies a meta-analysis is reported. Most of the reported meta-analyses evaluated a medical intervention (73%) and were preceded by a systematic review (83%). Only 54% of the meta-analyses reported also effect estimates of individual studies. Mostly (48%) a random effects model was chosen for data synthesis (Table 3).

**Table 3:** Results of included meta-analyses.

	Results from Peters et al. and Korevaar et al.		Results from this literature search		All results	
	Number absolute	Number in percentage*	Number absolute	Number in percentage	Number absolute	Number in percentage
**Meta-Analysis included**	93/266	35%	63/246	26%	156/512	31%
Meta-analysis evaluating a medical intervention	65/93	70%	49/63	78%	114/156	73%
Meta-analysis preceded by a systematic review	71/93	76%	59/63	94%	130/156	83%
Meta-analysis combines animal and human data	20/93	22%	8/63	13%	28/156	18%
**Number of included studies**						
Not reported	18/93	19%	16/63	25%	34/156	22%
<10	16/93	17%	11/63	18%	27/156	17%
10–50	46/93	50%	19/63	30%	65/156	42%
51–100	7/93	8%	9/63	14%	16/156	10%
>100	6/93	6%	8/63	13%	14/156	9%
**Methods for data synthesis**						
Not reported	37/93	40%	11/63	18%	48/156	31%
Fixed effects model	4/93	4%	1/63	2%	5/156	3%
Random effects model	38/93	41%	37/63	59%	75/156	48%
Both	14/93	15%	14/63	22%	28/156	18%
**Effect estimates of individual studies reported**	45/93	48%	39/63	62%	84/156	54%

*all percentages rounded to integral numbers.

### Citation of Systematic reviews and meta-analyses of animal studies on clinical research

Our search on Web of Science retrieved 337 articles, which cited the included 50 systematic reviews. Of these we excluded a total of 56 articles, because they could either not be classified as clinical studies and involved only animals (16/56), or were no original studies, but reviews (37/56), or letters (3/56). Thus, we included 281 articles reporting on 281 studies. Most of the included studies were randomized controlled trials or prospective cohort studies. The clinical studies referred to the preclinical research mainly to justify the current study or a future study (76%) (Table 4). Systematic reviews, which also included a meta-analysis have been cited more often (3 (0–73) (Mean (Min-Max))), than systematic reviews without meta-analysis by clinical studies (1 (0–32) (Mean (Min-Max))).

**Table 4:** Influence of systematic reviews of preclinical research on clinical research.

	Number absolute	Number in percentage*
**Type of clinical study** **		
Randomized controlled trials	87/281	31%
Non-randomized controlled trials	25/281	9%
Cross-over trials	2/281	1%
Uncontrolled prospective trials	6/281	2%
Retrospective cohort studies	42/281	15%
Prospective cohort studies	87/281	31%
Laboratory experiments with healthy human volunteers	6/281	2%
Cross-sectional surveys	1/281	0%
Case report/case series	26/281	9%
Guidelines	2/281	1%
Other	0/281	0%
**Use of citation** **		
Use of citation unrelated to animal studies	8/281	3%
Use of citation to provide at least partial justification for the study or a future study	213/281	76%
Use of citation to support or explain their findings	103/281	37%
Other	12/281	4%

*all percentages rounded to integral numbers.

**multiple selection possible.

### Comparison of included studies by Peters et al. and Korevaar et al. and studies included from this literature search

Updating the previous work of Peters et al. [5] and Korevaar et al. [6] we found a growing number of systematic reviews of animal studies. We compared the group of included systematic reviews by Peters et al. and Korevaar et al., which have been published between 1963 and 2010 to the systematic reviews included from our literature search published between 2009 and 2013 (Table 2 and Table 3). Looking at the methodological quality of the systematic reviews in the two groups the assessment of methodological quality and of heterogeneity remained similar, but dissemination bias is mentioned and assessed less often in the group by Peters et al. and Korevaar et al. than in the group of systematic reviews included from this literature search published between 2009 and 2013.

## Discussion

Korevaar et al. [6] found 163 systematic reviews of animal studies published between 2005 and 2010. We identified 246 systematic reviews between 2009 and January 2013. So the number of systematic reviews of animal studies roughly doubled in the last five years, similar to the trend already asserted by Korevaar et al. [6]. With the growing number of preclinical systematic reviews also the problem of low methodological quality and dissemination bias in systematic reviews of preclinical research is getting more attention [21], [22] and new efforts have been made to improve methodological quality, such as the CAMARADES initiative, or a newly developed RoB tool for animal intervention studies (SYRCLE's RoB tool) [11], [23]. Still, the methodological quality of preclinical systematic reviews and meta-analyses, especially the assessment of dissemination bias remains poor. But it seems that methodological quality improved, as PRISMA or Quality of Reporting of Meta-analyses (QUOROM) (14% vs. 3% (results from this literature search vs. results from included studies by Peters et al. and Korevaar et al.)) are more often mentioned and also seem to be followed more consequently as clear inclusion and exclusion criteria (65% vs. 56%), and the number of included studies (82% vs. 70%) is reported more often, and a list or flow diagram of the included studies (62% vs. 42%) is shown more often. The assessment of the methodological quality of included studies as well as the consideration and assessment of heterogeneity remained more or less unchanged. Over the last five years, the problem of dissemination bias has been recognized more widely, this we also showed in our data set, as dissemination bias has been considered (24% vs. 20%) and assessed (14% vs. 11%) more often [14]–[16]. A shift to more valid methods [24] such as funnel plot and statistical test (26% vs. 14%) can be noticed. Funnel plot is one of the simplest and most common used methods to detect dissemination bias in systematic reviews. It is a graphical method and its visual interpretation is subjective, and often there may be other reasons for funnel plot asymmetry than dissemination bias. Therefore, it is recommended to also run statistical test for funnel plot asymmetry to assess dissemination bias [10].

Preclinical research might even influence clinical research by informing the design of clinical studies. In this systematic review we showed that systematic reviews of animal studies are cited especially by clinical randomized controlled trials, which are considered the gold standard of clinical trials. Mostly, the citations are used to justify the conduct of the clinical trial (76%), but also to support or explain the findings (37%).

Our study has strengths and limitations. The strengths are that we used a comprehensive approach to identify systematic reviews of *in vivo* animal studies through a sensitive search strategy and inclusion of previously identified articles. We updated information on preclinical summaries. We also incorporated citation profiles to show the influence of animal research on clinical research. A limitation of our study is that our results might be affected by dissemination bias because we did not search any grey literature. Thus, this systematic review might miss a number of systematic reviews of preclinical research. We are therefore limiting the generalizability of our results about methodological quality and dissemination bias to the published systematic reviews of *in vivo* animal studies. Regarding the quality assessment one can assume that non-published systematic reviews might be of even lower quality and our results might be too positive. Regarding the influence on clinical studies non-published systematic reviews of animal studies might have less impact on clinical research, since they are not easily accessible. Furthermore, we arbitrarily selected only 50 systematic reviews and meta-analyses to assess their influence on clinical research. Thus, the citation rate might just show a trend of the influence of preclinical systematic reviews on clinical, above all since we did not measure the influence directly. This trend could be proven in further studies by assessing whether the objectives or the study design of the clinical studies is similar to the one of the preclinical study cited.

Research synthesis depends on high methodological quality of primary research. ARRIVE guidelines are helping to improve methodological quality and reporting of animal research [8]. Furthermore, it is important that all research results are accessible for systematic reviews in order to allow valid synthesis. Unfortunately, it has been shown that animal research often does not get published, and that the direction of results might be a reason for non-publication [14], [25]. In this study, there has been evidence for dissemination bias in 50% of systematic reviews, which assessed dissemination bias. Similarly Sena et al. also showed the presence and the impact of dissemination bias in systematic reviews of animal studies [25]. Thus if we agree that dissemination bias has an influence on the results of systematic reviews particularly of animal research, it can not only result in erroneous conclusions but might also lead to unsafe and unnecessary clinical research.

CAMARADES has already made a major step in improving systematic reviews of clinical research [11]. But in order to allow valid research synthesis, the availability of all research results is crucial. Therefore, the registration of animal studies before inception seems to be necessary [25], [26]. The registration of a clinical study, before the first participant has been included is required for publication; this should be applied to animal studies too. Since all animaly experiments must pass Institutional Animal Care and Use Committee or similar organizations for ethics approval they could play a crucial in the registration of animal studies and thus in the prevention of dissemination bias, as already suggested by ter Riet et al. [14].

### Conclusions and Implications

Over the years, the number of systematic reviews and meta-analyses of preclinical research has increased. In this systematic review, we showed that preclinical systematic reviews and meta-analyses influence clinical research and thus might influence even clinical care. Unfortunately, according to our data the quality of systematic reviews and meta-analyses of animal research still remains poor. Therefore, we strongly encourage every effort made to improve the methodology of systematic reviews and meta-analyses on preclinical research, such as CAMARADES or the registration of animal studies before inception [11].
